# Cecal microbiome profile altered by *Salmonella enterica*, serovar Enteritidis inoculation in chicken

**DOI:** 10.1186/s13099-018-0261-x

**Published:** 2018-08-03

**Authors:** Liying Liu, Lili Lin, Linna Zheng, Hui Tang, Xinzhong Fan, Nianguo Xue, Min Li, Min Liu, Xianyao Li

**Affiliations:** 1 College of Animal Science and Technology, Shandong Agricultural University, Tai’an, 271000 Shandong China; 2 College of Life Science, Shandong Agricultural University, Tai’an, 271000 Shandong China; 3 Shandong Provincial Key Laboratory of Animal Biotechnology and Disease Control and Prevention, Shandong Agricultural University, 61 Daizong Street, Tai’an, 271018 Shandong China

**Keywords:** Chicken, *Salmonella* Enteritidis, Cecal microbiota, 16S rRNA

## Abstract

**Background:**

*Salmonella enterica*, serovar Enteritidis (*S.* Enteritidis), an important zoonotic foodborne pathogen, can affect the microbiota of the chicken intestine and cause many enteric diseases, such as acute gastroenteritis. The gut microbiota contributes to the development and function of the host immune system and competes with pathogenic microbes. The interaction between *S.* Enteritidis and the host cecal microbiota is still not fully understood. We investigated the microbiome composition in both treated and control groups through 16S ribosomal RNA (rRNA) gene sequencing at 1, 3, 7, 14, 21, 28, and 35 days post-*S.* Enteritidis inoculation (dpi) in the current study.

**Results:**

Chao1 richness and Shannon diversity significantly increased with chicken development in both the treated and control groups (*P* < 0.05). The Chao1 index was significantly lower in the treated group than that in the control group at 14 dpi (*P* < 0.05). Phyla *Proteobacteria* and *Firmicutes* were most dominant at 1 and 3 dpi. *S.* Enteritidis inoculation influenced cecal microbiota mainly at 7 and 14 dpi. *S.* Enteritidis inoculation significantly altered the relative abundance of 18 genera at different time points (*P* < 0.05) with relative abundance significantly changed after 14 dpi. The abundance of those genera changed dramatically between 28 and 35 dpi in the treated group compared to control group. Positive correlations existed between *Bacillus* and *Blautia* and between *Coprococcus* and *Flavonifractor* following *S*. Enteritidis inoculation.

**Conclusions:**

Our results indicated that both development and *S.* Enteritidis have effect on chicken cecal microbiota profiles. *S.* Enteritidis inoculation in young chicks influences the cecal microbiota mainly at 7 and 14 dpi. The cecal microbiota exhibited immunity to *S.* Enteritidis inoculation at 28 dpi. These findings will provide basic knowledge of the role that chicken cecal microbiota play in response to *S.* Enteritidis inoculation.

**Electronic supplementary material:**

The online version of this article (10.1186/s13099-018-0261-x) contains supplementary material, which is available to authorized users.

## Background

*Salmonella enterica*, serovar Enteritidis (*S.* Enteritidis) is a common zoonotic pathogen that causes huge economic losses in the poultry industry. Humans can be infected with *S.* Enteritidis by consuming undercooked chicken products [1].

*S.* Enteritidis mainly colonizes the chicken cecum [2]. The cecal microbiome is primarily composed of *Firmicutes*, *Bacteroidetes* and *Proteobacteria* [3–5], *Bifidobacterium* provides endogenous sources of vitamins to enhance the chicken’s immune function [6]. Short-chain fatty acids produced by *Streptococcus faecalis* can reduce intestinal pH value and inhibit the growth of pathogens.

The intestinal microbiome matures as the chicken grows, developing rapidly from days [1–3, 7], and then tending to be stable. It has been reported that the early stages of hatching are the critical period for the establishment of chickens’ intestinal microbiota [8–10]. The complex intestinal microbial ecology, especially the microbiota of the gut developed in infancy, is closely intertwined with immune development [11]. Pathogen infection can affect host intestinal microbial composition. *S.* Enteritidis infection in young layer chicks significantly reduces the overall diversity of the microbiota population, promoting expansion of the Enterobacteriaceae family [12]. The gut microbiome in the ceca of pigs changed with *S. enterica*, serovar Typhimurium challenge [13].

Modern high-throughput deoxyribonucleic acid (DNA) sequencing approaches based on the 16S ribosomal RNA (rRNA) sequence—such as pyrosequencing, gene chip and single-strand conformation polymorphism—have been widely used to characterize the chicken gut microbiome [12, 14–16]. This has sped up understanding of the structural composition of intestinal microbiota as well as the interaction between these microorganisms and their host [17]. We conducted the current study to assess the diversity of the chicken cecal microbiome induced by *S.* Enteritidis inoculation and to provide a scientifically theoretical basis of interaction between pathogens and gut microbiota.

## Methods

### Animal inoculation

We used Jining Bairi chicken, a regional Chinese breed, in the current study. All chickens were provided by Shandong Bairi Chicken Breeding Co., Ltd. (Shandong, China). We purchased the *S.* Enteritidis strain (CVCC3377) used for the inoculation from the China Veterinary Culture Collection Center, Beijing.

We collected meconium from each individual chicken and checked it for *S.* Enteritidis negativity using the plating method. In total, we randomly assigned 168 two-day-old *S.* Enteritidis–negative chickens into 2 groups of 84 chickens each treated (trt) and control (con) groups and raised them in 2 separate incubators with the same environmental conditions and with access to food and water ad libitum. Each chicken in the treated group was orally inoculated with 0.3 ml 10^9^ colony-forming units (cfu)/ml *S.* Enteritidis inoculant, while chickens in the control group were mock-inoculated with the same amount of sterile phosphate buffer saline (PBS). Twelve chickens from each of the treated and control groups were euthanized by cervical dislocation for sample collection at 1, 3, 7, 14, 21, 28 and 35 days post-inoculation (dpi). All animal procedures were approved by Shandong Agricultural University Animal Care and Use Committee.

### Enumeration of *S*. Enteritidis in cecal content

We collected fresh cecal content from 1 cecal pouch in each chicken, weighed it, put it on ice and sent it to laboratory for *S.* Enteritidis enumeration. We then collected the cecal content from another cecal pouch in the same chicken and froze it at − 20 °C for DNA extraction. To assess the amount of *S.* Enteritidis in the cecal content from each individual chicken, we diluted the samples, plated them on *Salmonella*–Shigella agar and incubated them for 24 h at 37 °C. Each sample was processed in triplicate.

### DNA extraction from cecal content and polymerase chain reaction (PCR) amplification of the 16S rRNA gene

At 1 and 3 dpi, cecal content from 3 randomly selected chickens were mixed with equal amount to get enough sample for DNA extraction. In total, 3 mixed cecal content samples were obtained from treated group and 3 from control group at 1 and 3 dpi, respectively. At each time point from 7 to 35 dpi, individual cecal content was randomly selected and used for DNA extraction. Genomic DNA was extracted from 500 mg cecal content using a fecal genomic DNA extraction kit (CWBio, Beijing, China). We examined DNA integrity by agarose gel electrophoresis and measured DNA concentration and purity using a DS-11 spectrophotometer (DeNovix, Wilmington, Delaware, US). We stored the qualified DNA samples at − 20 °C for further analysis.

We performed PCR amplification with forward (5′-ACTCCTACGGGAGGCAGCA-3′) and reverse (5′-GGACTACHVGGGTWTCTAAT-3′) primers targeting the V3 and V4 segments of the 16S rRNA gene. PCR conditions were set for initial denaturation at 95 °C for 5 min, followed by 25 cycles of 95 °C for 30 s, 50 °C for 30 s and 72 °C for 40 s, with a final extension step at 72 °C for 7 min. We submitted the amplicons to Biomarker Technologies Co., Ltd (Beijing, China) to generate 250 paired-end reads on the MiSeq sequencing platform (Illumina, Inc., San Diego, California, US). The data has been deposited into Sequence Read Archive (National Center for Biotechnology Information, National Institutes of Health, Bethesda, Maryland, US) [18, 19].

### 16S rRNA gene sequencing and data analysis

FLASH [20] was used to merge paired end reads before assembly. Trimmomatic [21] was used to remove adapters, low-quality sequences and reads shorter than 36 bases. We predicted the chimeric sequences and excluded them from the analysis [22] to get high-quality tag sequences. Similar sequences were clustered into operational taxonomic units (OTU) at a 97% identity threshold using UCLUST software version 1.2.22 (https://www.drive5.com/) [23]. We filtered the OTUs using 0.005% of the number of all sequences as thresholds [24].

We analyzed the alpha diversity metrics, including Chao1 (richness estimate) and Shannon and Simpson diversity indices, using mothur software version 1.30 (mothur project, Department of Microbiology & Immunology, University of Michigan, Ann Arbor, Michigan, US) [25]. Beta diversity was analyzed using unweighted UniFrac distances [26] followed by principal-component analysis (PCA). We generated a cluster of all samples based on unweighted UniFrac distances using the heatmap function in R 3.4 software (https://www.r-project.org/), constructed a polygenetic tree of all samples using Molecular Evolutionary Genetics Analysis (MEGA) 7 [27] software and identified cladograms with statistically significant taxonomic differences between the groups. In our linear discriminant analysis with effect size (LEfSe; http://huttenhower.sph.harvard.edu/galaxy), we used a linear discriminant analysis (LDA) value of 4.0 and effect size threshold of 2. We performed our redundancy analysis (RDA) at the bacterial-group level.

### Statistical analysis

We evaluated OTUs and alpha and beta diversity between the 2 groups at each time point using unpaired *t*-tests. We determined alpha and beta diversity metrics across different time points for both groups using analysis of variance (ANOVA). We used the Bonferroni method to compare multiple means. *P* < 0.05 was considered significant.

## Results

### Data assessment and OTU assignment

We obtained a total of 6,348,060 16S rRNAs (V3+V4 regions) with an average of 151,144 reads per samples. After assembly, we had 77,085–170,126 raw tags per sample. After filtering and quality checking, which was required for more than 60% of raw tags, there were 50,294–115,062 clean tags, to which we assigned taxonomy. We then mapped the reads to generate 13,093 OTUs that could be grouped into 544 unique OTUs. The number of OTUs in each sample ranged from 59 to 461 with an average of 311 across samples (Additional file 1).

### Microbial diversity changed temporally

**Fig. 1:**
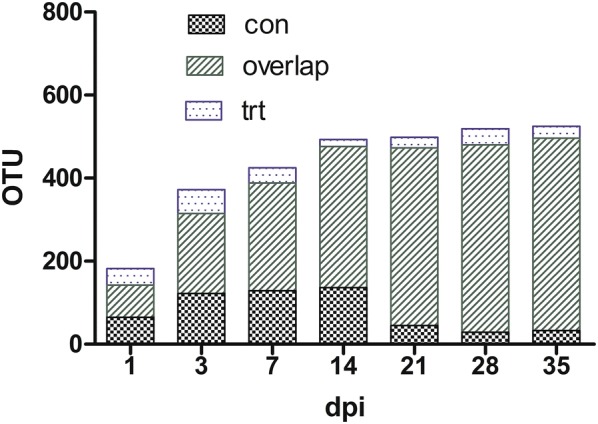
Number of OTUs in each group. *OTUs* operational taxonomic units, *dpi* day post-inoculation, *con* control group, *trt* treated group

OTUs detected in at least 1 sample from one group were counted into the number of OTUs in that group (Fig. 1). In the control group, there were 142, 315, 388, 476, 473, 480 and 496 OTUs obtained at 1, 3, 7, 14, 21, 28 and 35 dpi, respectively. In the treated group, there were 117, 250, 296, 357, 453, 490 and 492 OTUs identified at 1, 3, 7, 14, 21, 28 and 35 dpi, respectively. There were 77, 193, 259, 340, 428, 451 and 463 OTUs that overlapped between both groups at 1, 3, 7, 14, 21, 28 and 35 dpi, respectively (Fig. 1).

**Fig. 2:**
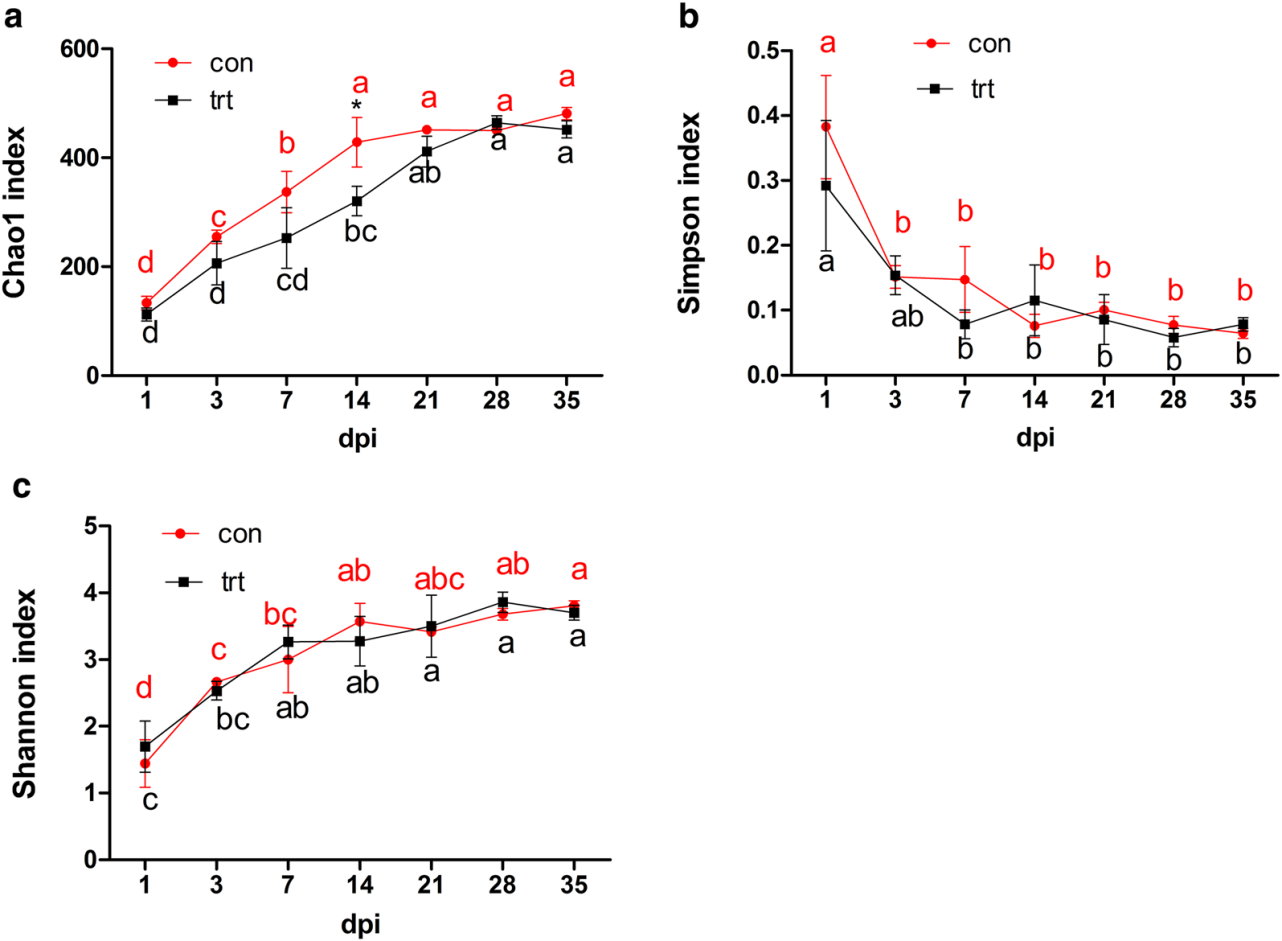
Cecal microbial alpha diversity at different time points in treated and control groups. (**a**) Chao1 index in control and treated groups; (**b**) Simpson index in the control and treated groups; (**c**) Shannon index in the control and treated groups. *trt* treated group, *con* control group

We compared alpha diversity metrics across 7 different time points within both groups (Fig. 2). In the control group, Chao1 richness increased significantly between 1 dpi (133.11) and 14 dpi (428.53; *P *< 0.05), but not significantly between 14 and 35 dpi. Simpson diversity at 1 dpi (0.38) was significantly higher than at other time points, while Shannon diversity at 1 dpi was significantly lower than at other time points (both *P *< 0.05). In the treated group, Chao1 richness increased between 1 dpi (112.25) and 28 dpi (464.41; *P* < 0.05) and was significantly lower at 1 and 3 dpi than that at 14, 21, 28 or 35 dpi (*P* < 0.05). Simpson diversity was significantly higher at 1 dpi (0.29) than that at 7, 14, 21, 28 or 35 dpi, while Shannon diversity was significantly lower at 1 and 3 dpi than that at 21, 28 or 35 dpi (both *P* < 005).

**Fig. 3:**
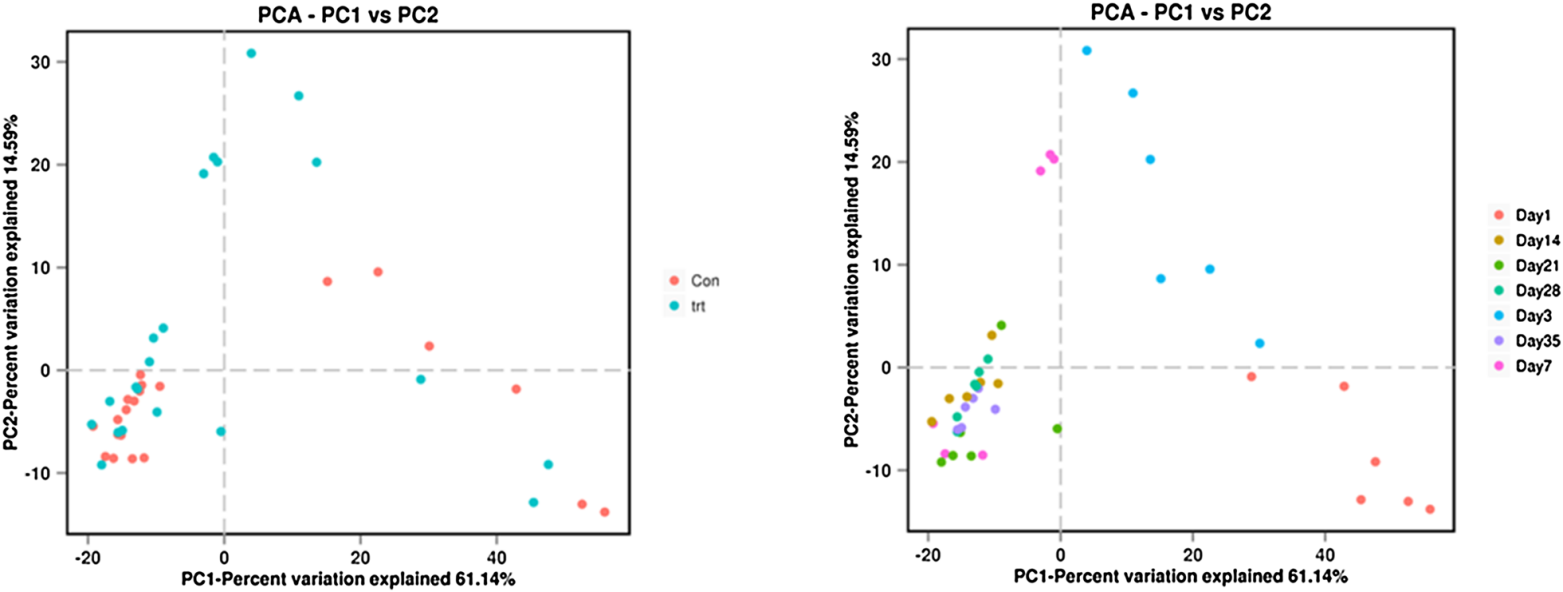
PCA of unweighted UniFrac distances as a measure of beta diversity across samples. Each point represents 1 sample. Samples in the same group are labeled the same color. *trt* treated group, *con* control group

**Fig. 4:**
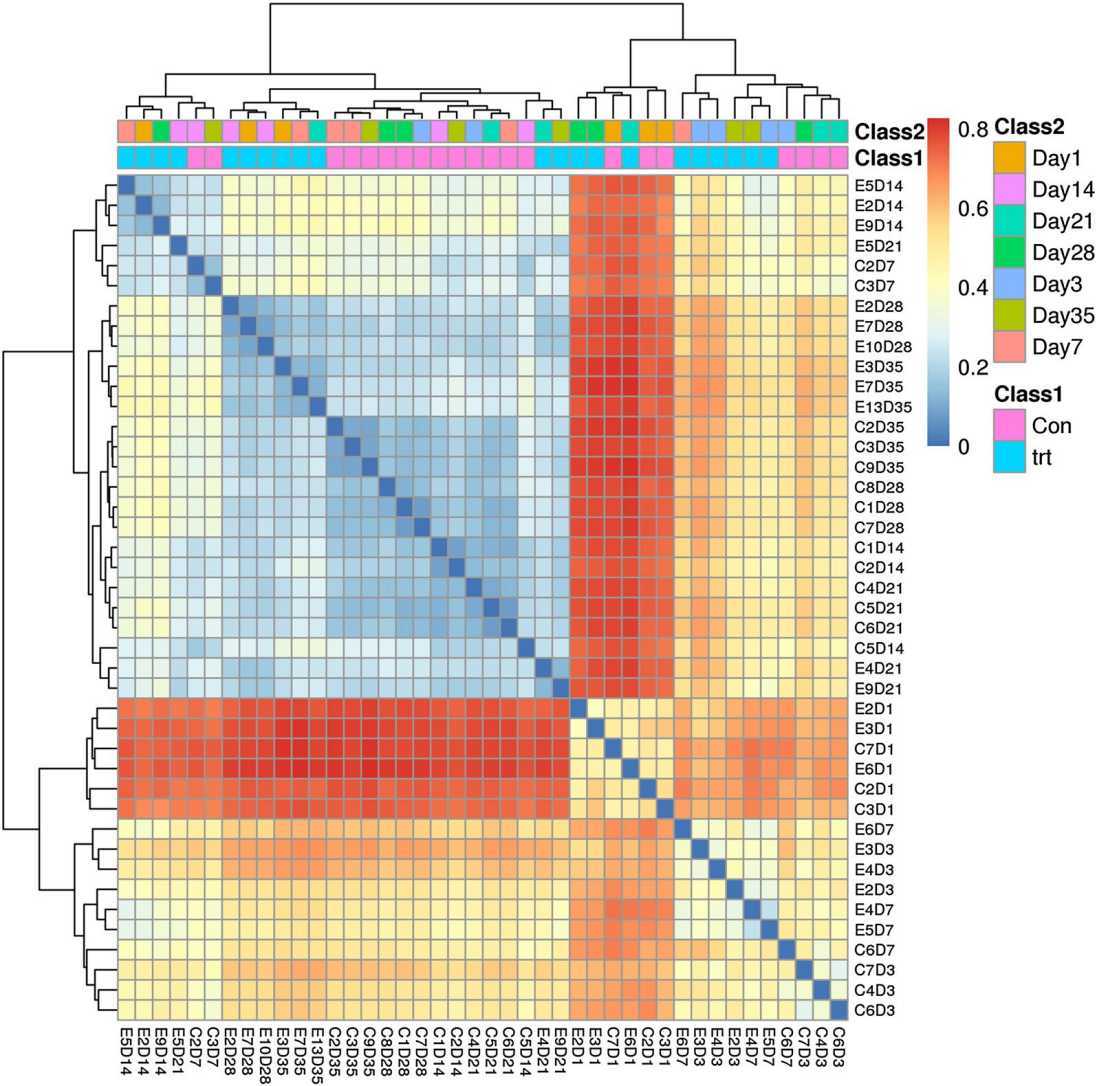
Heatmap across all samples based on unweighted UniFrac distances. C2D1, sample C2 in control group at 1 dpi

**Fig. 5:**
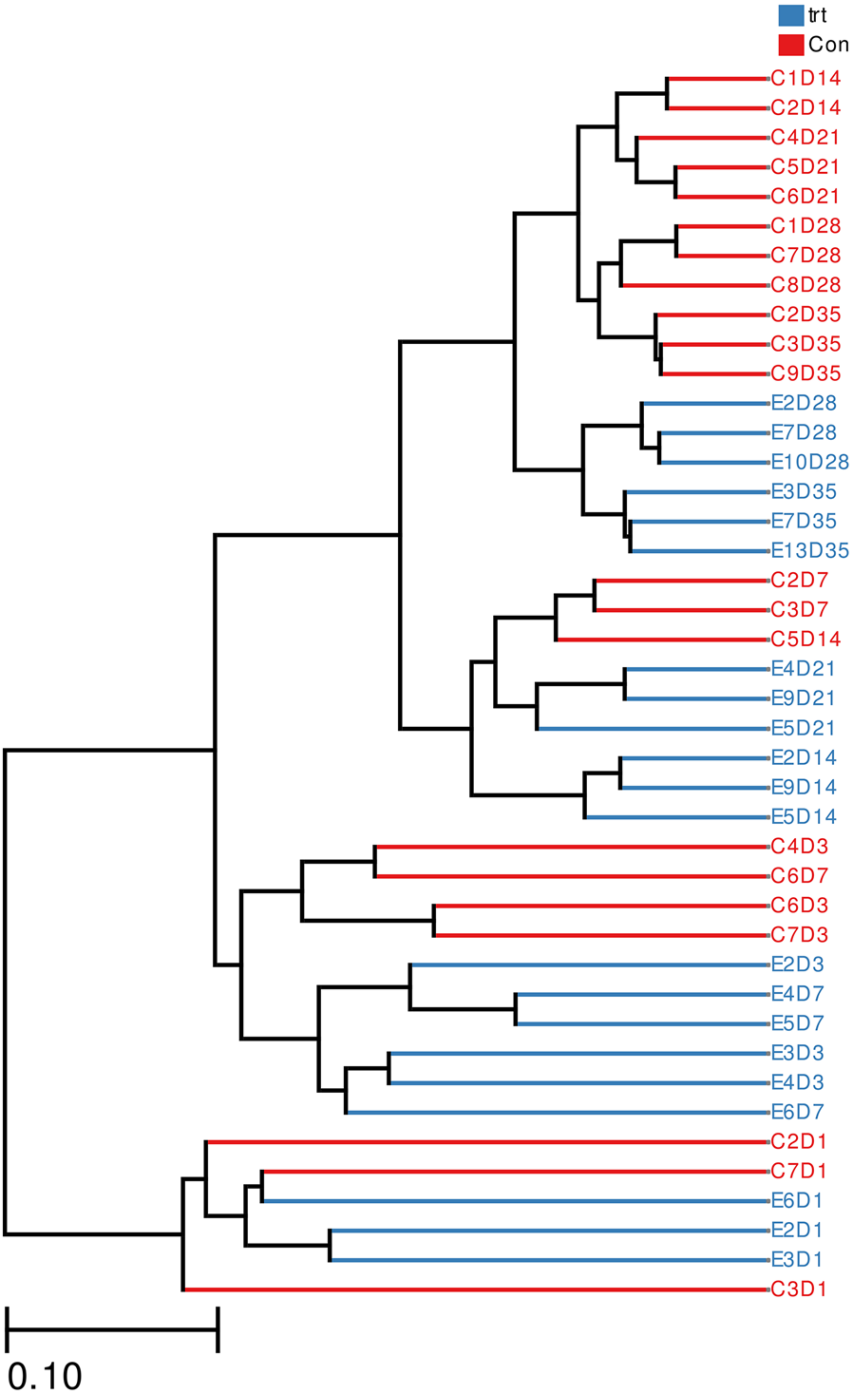
Phylogenetic tree across all samples based on unweighted UniFrac distances. C2D1, sample C2 in control group at 1 dpi

We used unweighted UniFrac distances for the PCA to determine if the samples had grouped into distinct clusters due to beta diversity (Fig. 3). Results showed that samples taken from each group at 1, 3, and 7 dpi formed distinct clusters. Samples taken at 14, 21, 28 and 35 dpi formed a single cluster regardless of *S.* Enteritidis inoculation. Heatmap results based on unweighted UniFrac distances showed that all samples were grouped into 3 clusters: samples taken at 3 and 7 dpi, in which treated and control samples were clearly separated; samples taken at 1 dpi; and samples taken at 14, 21, 28 and 35 dpi, which were divided distinctly between both groups (Fig. 4). Phylogenetic-tree results showed that samples taken at 1 dpi formed one distinct node separated from the other samples. Samples at 3 and 7 dpi were separated from samples at 14, 21, 28 and 35 dpi. Samples in the treated and control groups at 14, 21, 28 and 35 dpi were divided into two groups (treated and control groups). In each group, samples taken at 14 and 21 dpi clustered together, as did samples taken at 28 and 35 dpi (Fig. 5).

### Microbiome composition changed temporally

**Fig. 6:**
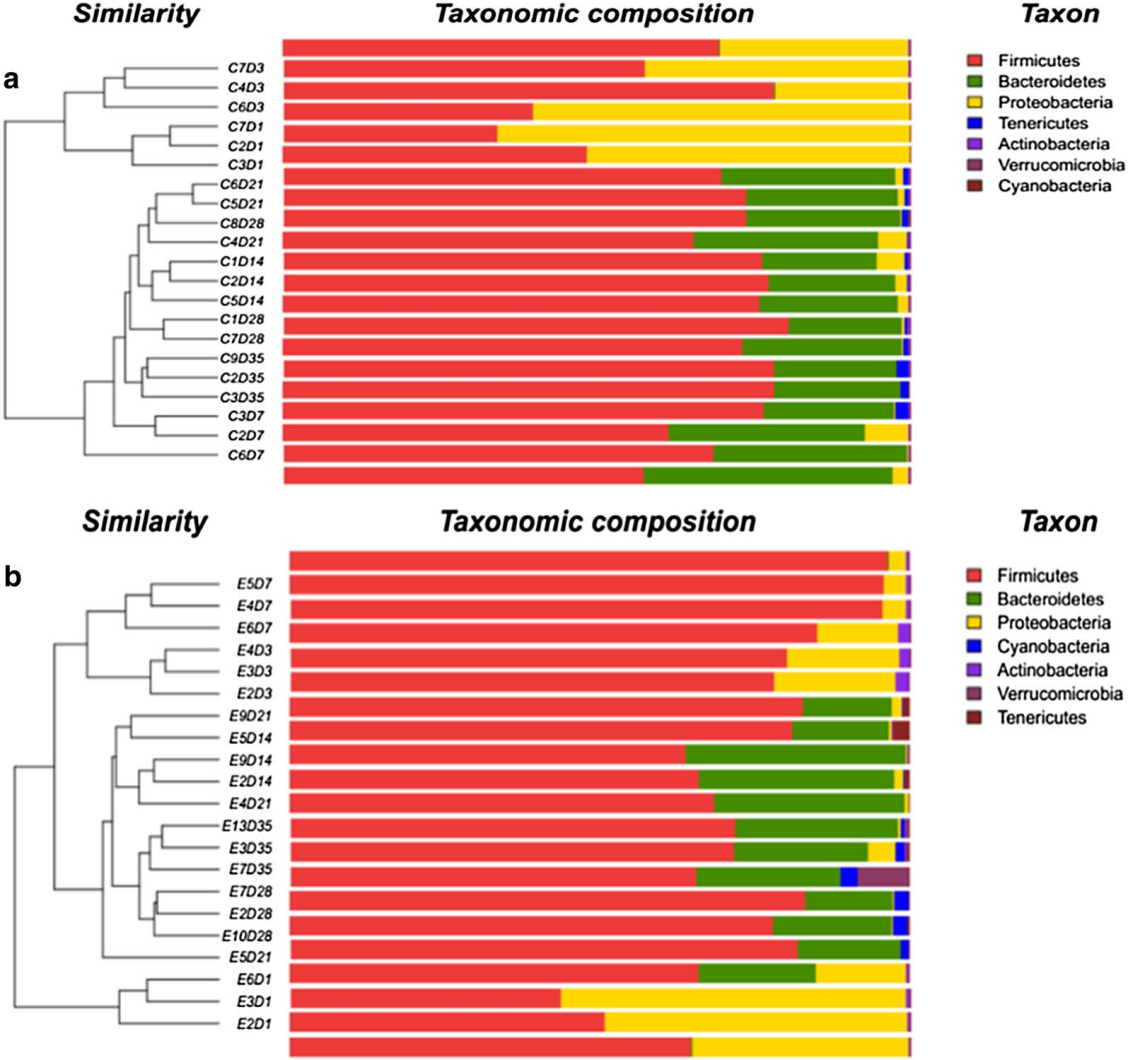
Differential abundances of cecal microbial communities on phylum level across samples within treated or control group. Top 10% most-abundant phyla were shown in the figure. (**a**) abundances of microbial community across samples within control group. (**b**) aduncances of microbial community across samples within treated group

**Fig. 7:**
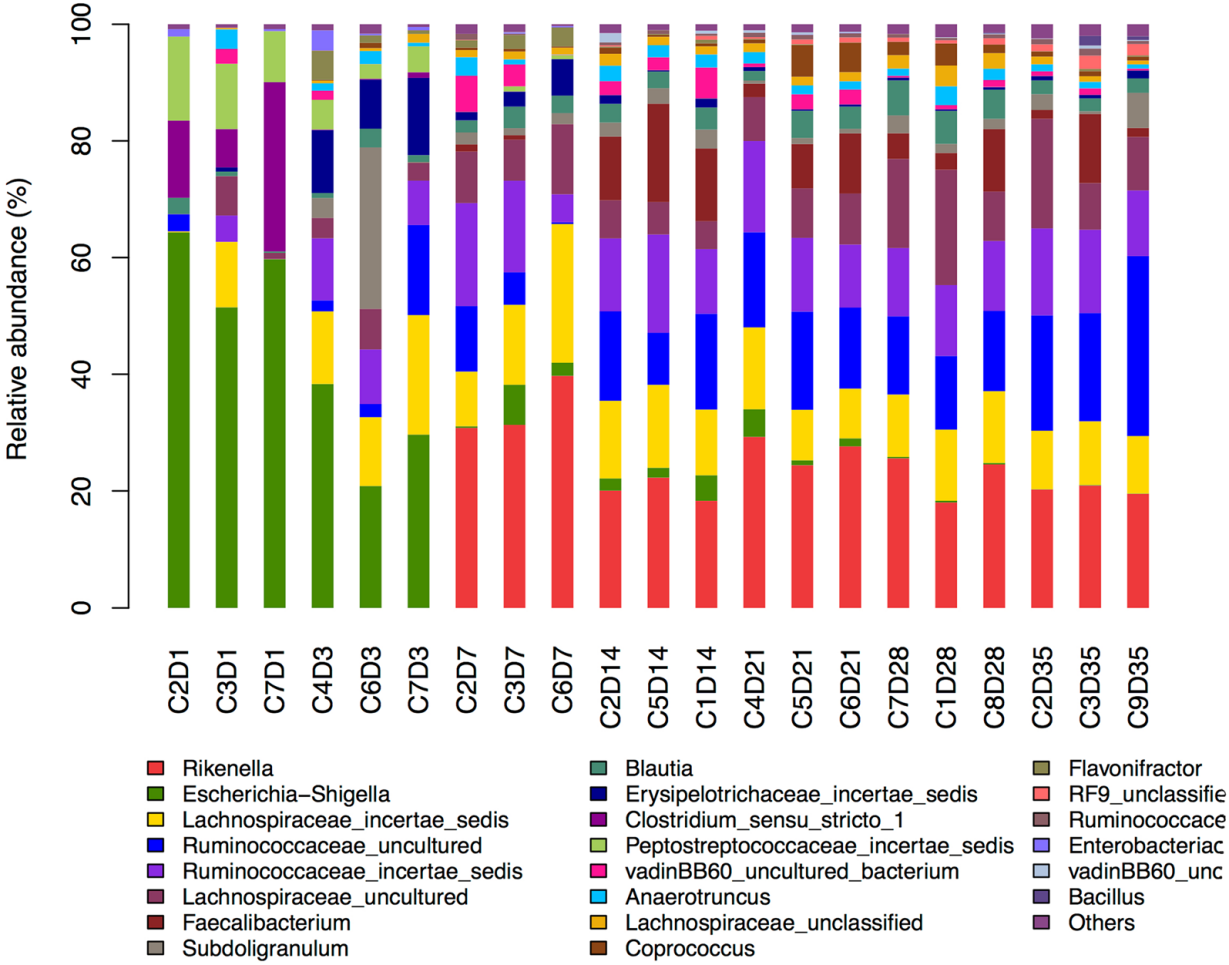
Differential abundance of cecal microbial communities on genus level across samples within control group. Top 10% most-abundant genera were shown in the figure

We analyzed microbiota composition across different time points within both groups. Compositional differences of the 7 most abundant phyla were shown in Fig. 6. Differential abundance of phyla detected across different time points within the control group showed that phyla *Proteobacteria* and *Firmicutes* were most dominant at 1 and 3 dpi. The most abundant phyla at 7, 14, 21, 28 and 35 dpi were *Firmicutes* and *Bacteroidetes* (Fig. 6a). Differential abundance of genera across different time points in the control group showed that *Escherichia*–*Shigella*, Ruminococcaceae *incertae sedis* and Peptostreptococcaceae *incertae sedis* were abundant at 1 dpi; *Escherichia*–*Shigella*, Lachnospiraceae *incertae sedis* and Erysipelotrichaceae *incertae sedis* at 3 dpi; and *Rikenella*, R. *incertae sedis*, L. *incertae sedis* and uncultured Ruminococcaceae from 7 to 35 dpi (Fig. 7).

**Fig. 8:**
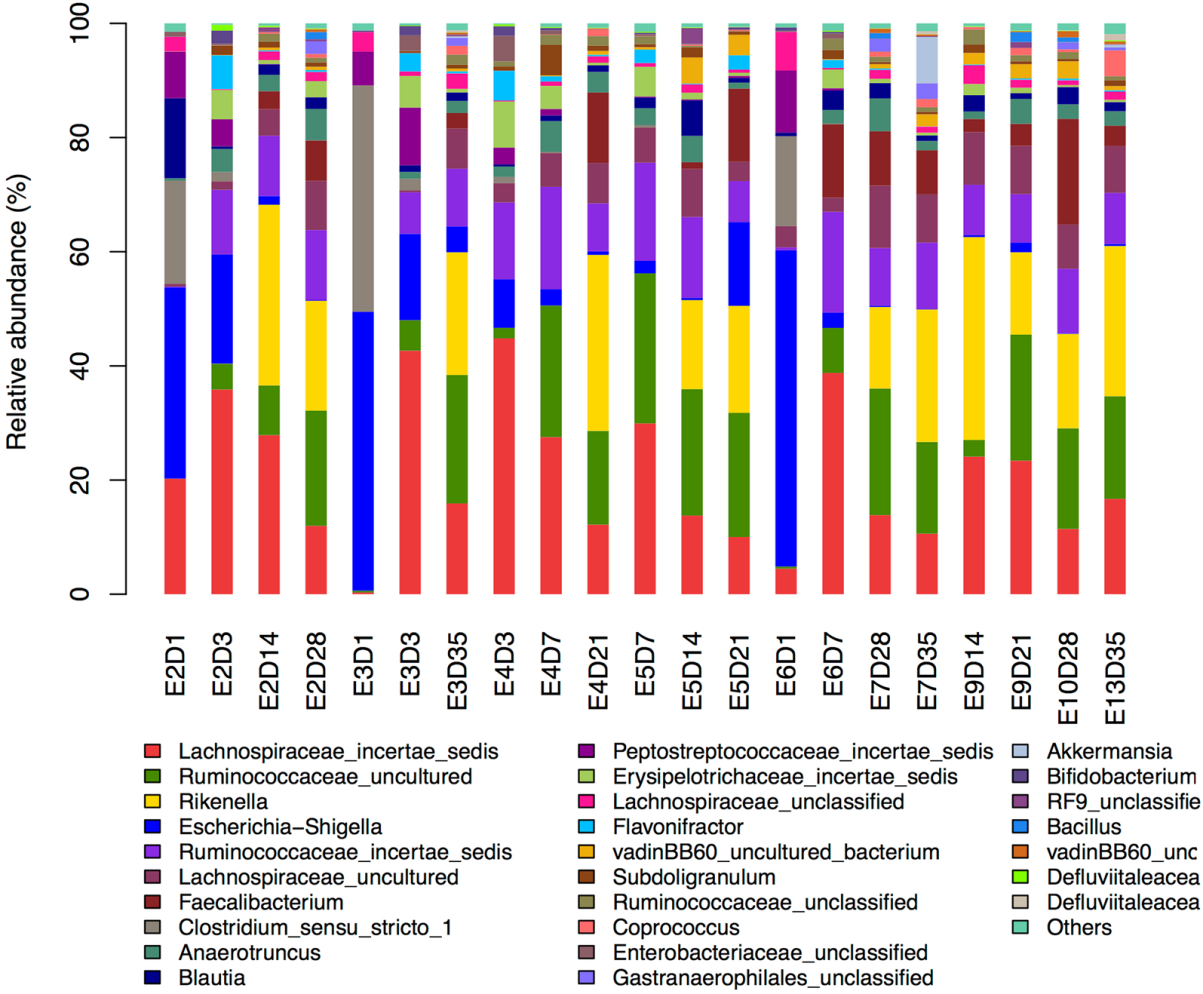
Differential abundance of cecal microbial communities on genus level across samples within treated group. Top 10% most-abundant genera were shown in the figure

We further analyzed microbiota composition across different time points within the treated group, then compared relative abundance of compositional differences in the 7 most abundant phyla across different time points. Differential abundance in phyla detected in the different time points showed that phyla *Proteobacteria* and *Firmicutes* were most dominant at 1 and 3 dpi. The most abundant phyla at 14, 21, 28 and 35 dpi were *Firmicutes* and *Bacteroidetes*. *Firmicutes* was the most abundant phylum at 7 dpi (Fig. 6b). Differential abundance in genera across different time points within the treated group showed that *Escherichia*–*Shigella* and *Clostridium* sensu stricto *1* were abundant at 1 dpi; Lachnospiraceae *incertae sedis*, *Escherichia*–*Shigella* and *Blautia* at 3 dpi; L. *incertae sedis*, uncultured Ruminococcaceae, R. *incertae sedis* and *Faecalibacterium* at 7 dpi; and L. *incertae sedis*_uncultured Ruminococcaceae, *Rikenella* and *Faecalibacterium* at 35 dpi (Fig. 8).

**Fig. 9:**
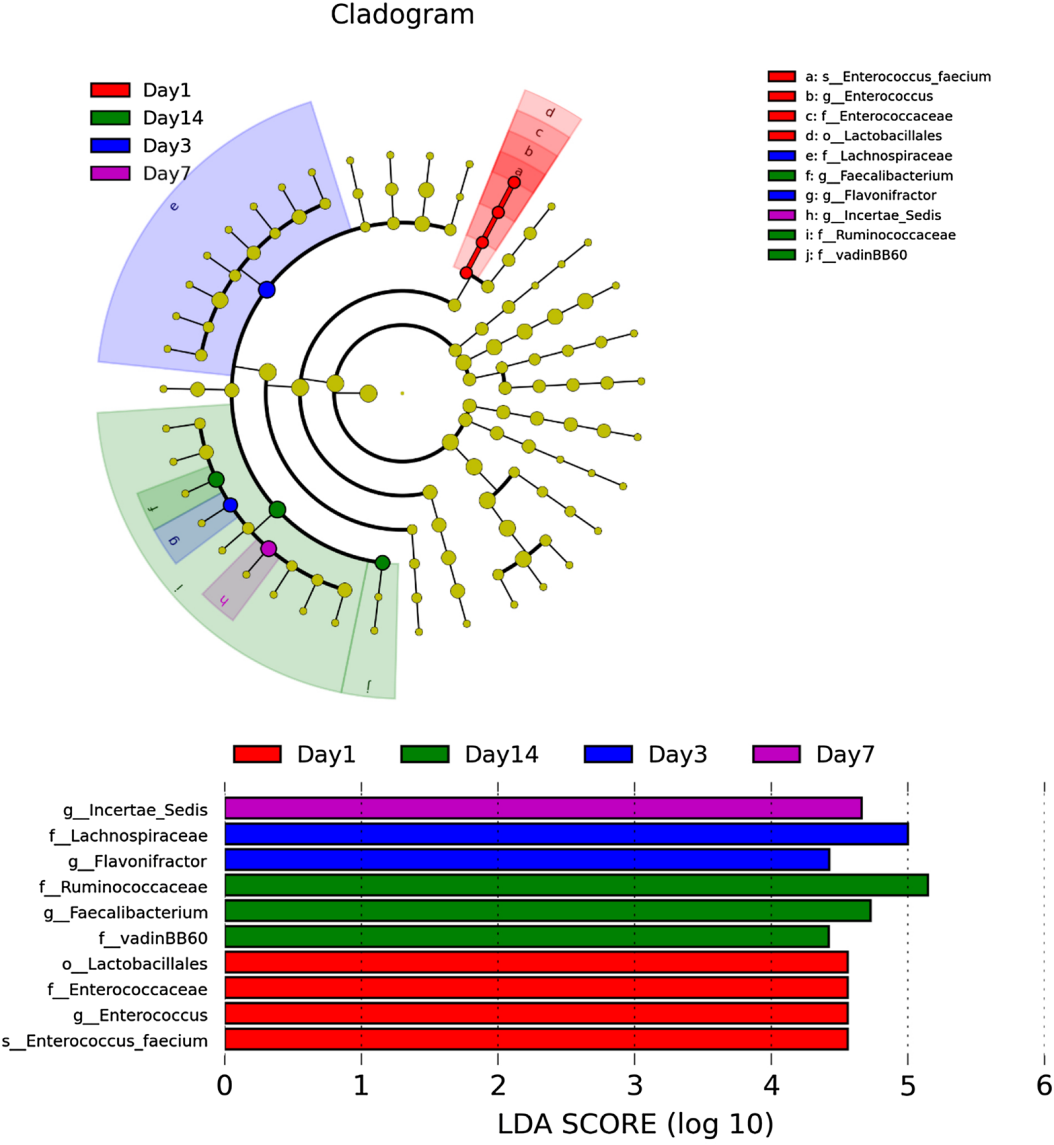
Taxonomic cladogram generated from LEfSe analysis showing significant difference in microbiota profile across different time points

We then analyzed microbiota composition across different time points using LEfSe and observed abundant microbiota composition at 1, 3, 7 and 14 dpi (Fig. 9). *Enterococcus faecium* (order Lactobacillales, family Enterococcaceae) was dominant at 1 dpi; genus *Flavonifractor* (family Lachnospiraceae) at 3 dpi; genus L. *incertae sedis* dominant at 7 dpi; and families Ruminococcaceae and VadinBB60 and genus *Faecalibacterium* at 14 dpi.

### Differential microbial composition and abundance between treated and control groups

**Fig. 10:**
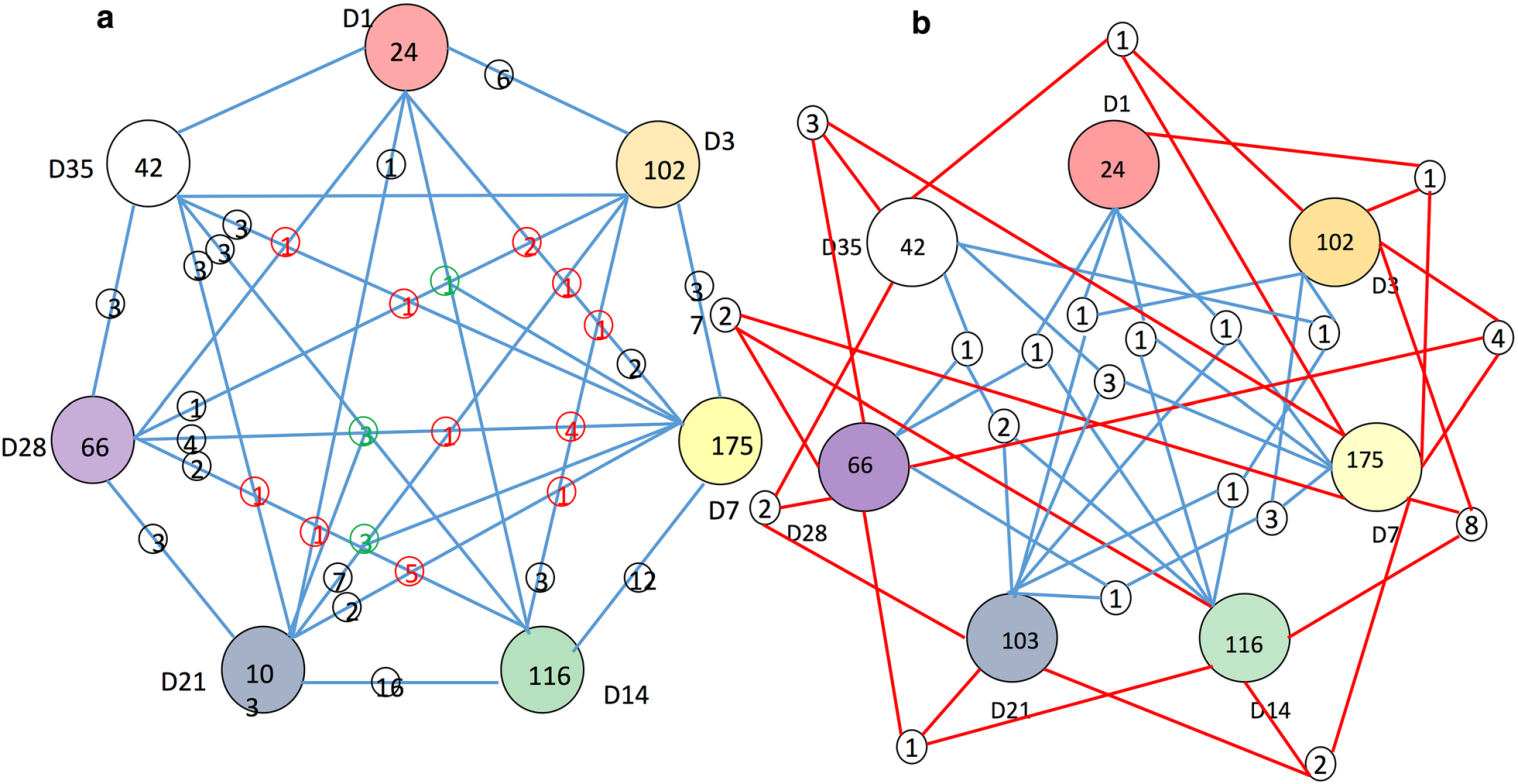
Overlapped significantly different OTUs (SDOs) between trt and con groups across different time points. D1, D3, D7, D14, D21, D28 and D35 represent trt vs. con at 1, 3, 7, 14, 21, 28 and 35 dpi, respectively. D1, D3, D7, D21, D28 and D35 values represented numbers of unique SDOs in each comparison. (**a**) Number of SDOs across 2, 4 and 5 comparisons. Value in black on the line represented number of SDOs between 2 comparisons. Value in red on the node represented number of SDOs across 4 comparisons. Value in green on the node represented number of SDOs across 5 comparisons. (**b**) Number of SDOs across 3 comparisons

Number of OTUs differed between the two groups at each time point (Fig. 10):

- There were 24, 102, 175, 116, 103, 66 and 42 unique OTUs that significantly differed between the two groups at 1 (trt/con1), 3 (trt/con3), 7 (trt/con7), 14 (trt/con14), 21 (trt/con21), 28 (trt/con28) and 35 dpi (trt/con35), respectively (*P* < 0.05).
- There were 37, 12 and 16 significantly different OTUs overlapping between trt/con3 and trt/con7, trt/con7 and trt/con14, and trt/con14 and trt/con21, respectively.
- Five significantly different OTUs overlapped across trt/con3, trt/con7 and trt/con14.
- Three significantly different OTUs overlapped among trt/con3, trt/con7, trt/con14, trt/con21 and trt/con28.
- Three significantly different OTUs overlapped among trt/con7, trt/con14, trt/con21, trt/con28 and trt/con35.

We compared alpha diversity between the 2 groups at each time point following *S.* Enteritidis inoculation (Fig. 2). Chao1 richness in the control group was significantly higher than that that in the treated group at 14 dpi (*P* < 0.05). We compared beta diversity across different time points within either group using unweighted UniFrac distances followed by PCA analysis to determine relative abundance of microorganisms. Microbial composition in the control group across 7 time points was separated into 3 different clusters: samples at 1 dpi; samples at 3 dpi; and samples at 7, 14, 21, 28 and 35 dpi (Additional file 2). Microbial composition in the treated group across 7 time points was separated into 2 different clusters; samples at 28 and 35 dpi were closely clustered together, and other samples were scattered (Additional file 3).

We then analyzed the differentially abundant genera between the treated and control groups at each time point (Additional file 4). Results showed that 18 genera differed significantly between the treated and control groups at 7 different time points. The groups did not significantly differ in genera at either 1 or 21 dpi. At 3 dpi, *Bifidobacterium* and *C.* sensu stricto *1* were more abundant in the treated group than in the control group, but *Intestinimonas* was significantly less abundant in the treated group than in the control group (*P* < 0.05). At 7 dpi, *Bifidobacterium* was more abundant in the treated group than in the control group, but *Rikenella* and *Coprococcus* were less abundant in the treated group than in the control group (*P* < 0.05). At 14 dpi, *Anaerostipes* was significantly more abundant in the treated group than in the control group, but *Faecalibacterium* and *Subdoligranulum* were less abundant in the treated group than in the control group (*P* < 0.05).

The abundance of 7 genera—*Bacillus*, *Enterococcus*, *Anaerostipes*, *Blautia*, *Shuttleworthia*, *Flavonifractor* and *Intestinimonas*—was significantly different between the treated and control groups at 28 dpi. Of those genera, *Bacillus, Enterococcus*, *Anaerostipes*, *Flavonifractor* and *Intestinimonas* were more abundant in the treated group than in the control group, but *Blautia* and *Shuttleworthia* were less so in the treated group than in the control group (*P *< 0.05). Five genera significantly differed in abundance between the treated and control groups at 35 dpi. Four of the 5—Lachnospiraceae *incertae sedis*, *Anaerostipes*, *Blautia* and *Hydrogenoanaerobacterium*—were less abundant in the treated than in the control group, but *Anaerotruncus* was more abundant in the treated group than in the control group (*P* < 0.05). *Salmonella* was detected in all chickens in the treated groups.

**Fig. 11:**
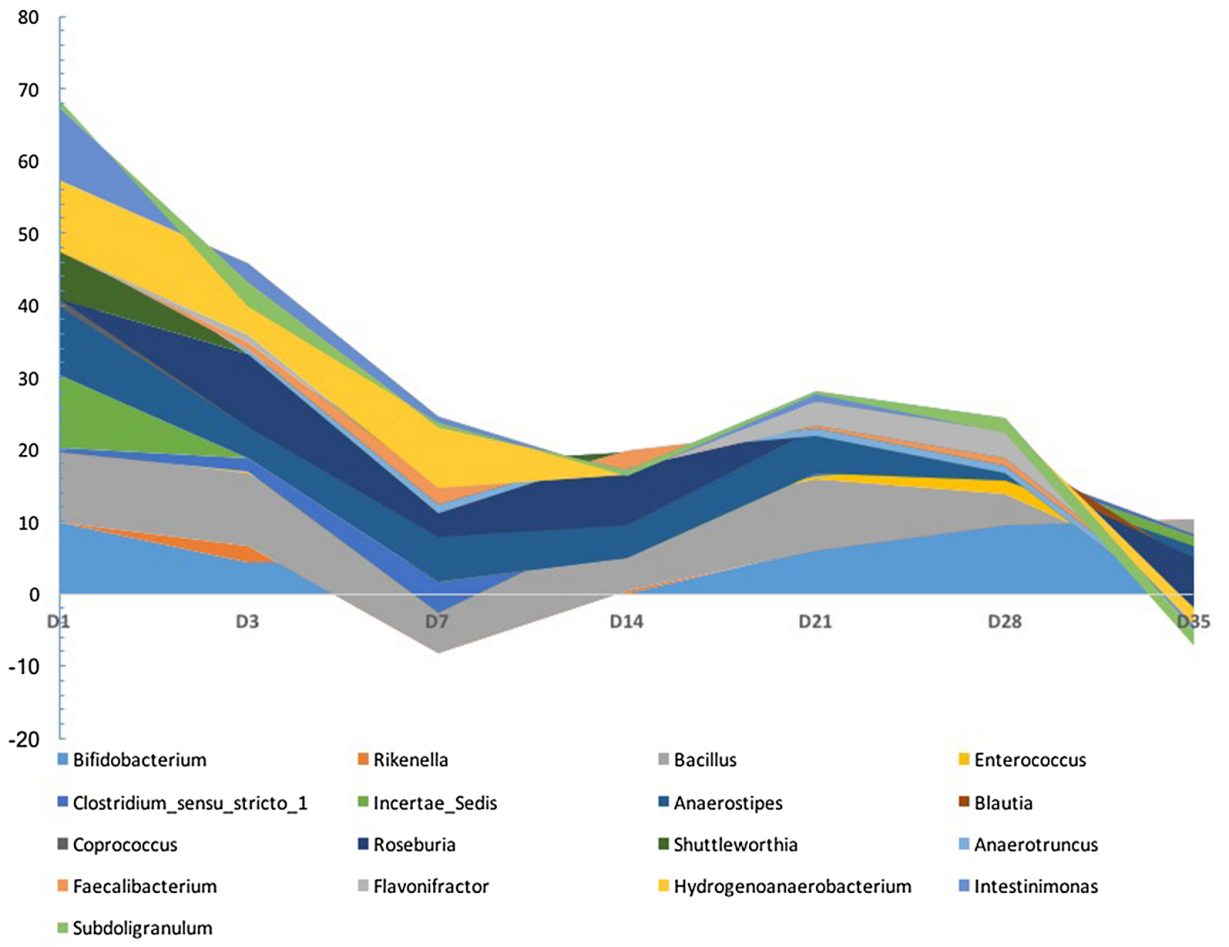
Relative abundance of significantly different genera between treated and control groups. The log2-transformed relative abundances of significantly different genera were used for plotting. The biggest value of 10 was assigned to the genus uniquely detected in either treated or control. *Salmonella* was detected only in treated group across different time points

We analyzed the relative abundance of each genus between the two groups, which was shown in Fig. 11. The V-shape in that figure illustrated the dramatic change in relative abundance for all genera in the treated group from 1 to 35 dpi. Relative abundance of *Bacillus* dropped at 7 dpi; that of many genera reversed itself between 28 and 35 dpi.

Differences in microbial community abundance (at the genus level) between the 2 groups across several time points were shown in Table 1. We observed significantly different abundance of 8 genera between the groups (*P* < 0.05). *Salmonella* was detected only in the treated group. *Bifidobacterium* was significantly more abundant in the treated group than that in the control group, while we detected *Cellulosilyticum* only in the control group (*P* < 0.01). *Anaerotruncus* and *Epulopiscium* were significantly more abundant in the treated group than in the control group, while *Roseburia*, *Shuttleworthia* and *Subdoligranulum* were significantly less abundant in the treated group (*P* < 0.05).

**Table 1:** Differences in microbiome between control and treated groups

Genus	Control	Treated	*P* value
*Bifidobacterium*	2.28E−04 ± 9.68E−05	3.81E−03 ± 1.43E−03	0.001
*Cellulosilyticum*	1.42E−04 ± 1.42E−04	0.00E+00 ± 0.00E+00	0.001
*Salmonella*	–	5.91E−04 ± 2.67E−04	
*Shuttleworthia*	1.49E−03 ± 3.81E−04	4.68E−04 ± 1.63E−04	0.017
*Anaerotruncus*	1.58E−02 ± 2.24E−03	2.68E−02 ± 3.87E−03	0.02
*Epulopiscium*	2.07E−06 ± 1.47E−06	4.53E−04 ± 2.42E−04	0.021
*Subdoligranulum*	2.98E−02 ± 1.28E−02	9.62E−03 ± 2.47E−03	0.031
*Roseburia*	6.68E−04 ± 2.59E−04	1.07E−04 ± 1.06E−04	0.043

**Fig. 12:**
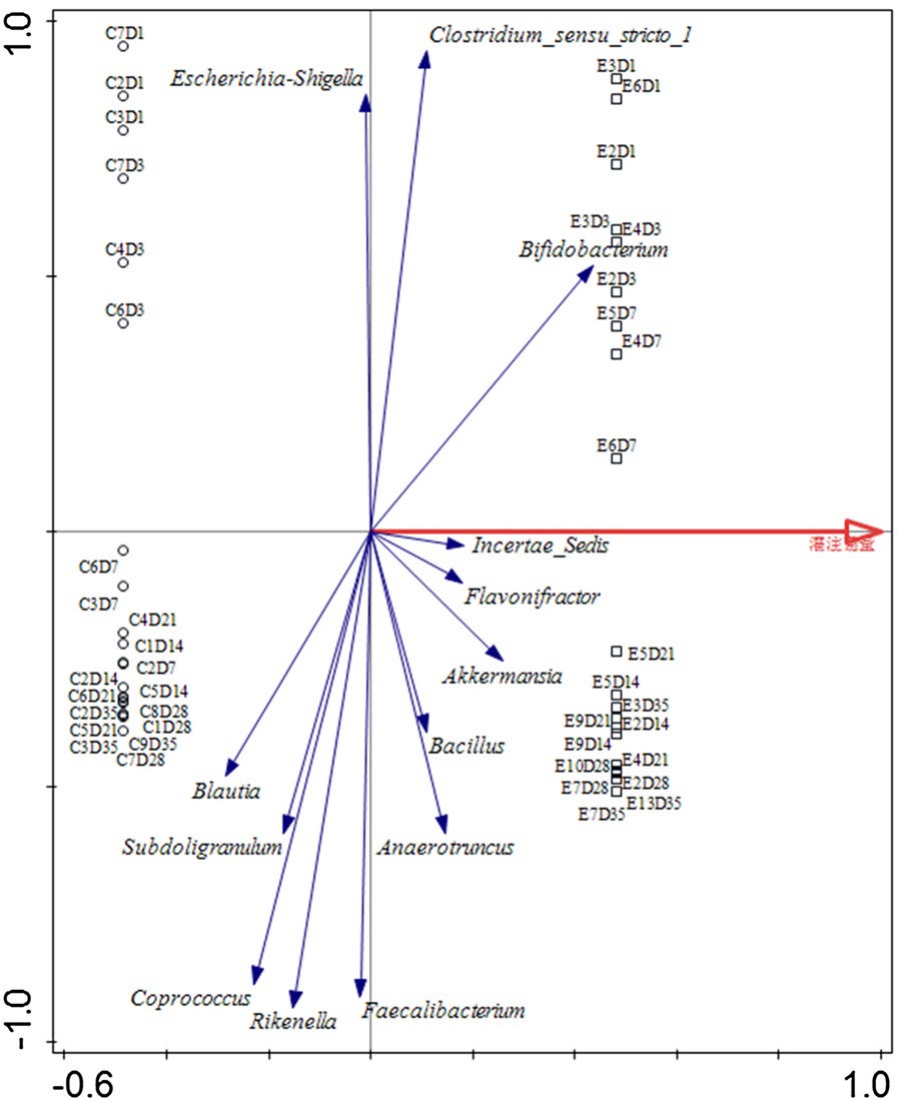
RDA of the relationship between *S.* Enteritidis inoculation and relative abundance of microbiota at genus level. Arrows indicate the direction and magnitude of variables

RDA results showed that *S.* Enteritidis affected chicken cecal microbiota (Fig. 12). Potential major genera driving community differentiation included *Escherichia*–*Shigella*, *C.* sensu stricto *1*, *Bifidobacterium*, L. Lachnospiraceae *incertae sedis*, *Flavonifractor*, *Akkermansia*, *Bacillus*, *Anaerotruncus*, *Faecalibacterium*, *Rikenella*, *Coprococcus*, *Subdoligranulum* and *Blautia*. *Bifidobacterium* was positively correlated with L. *incertae sedis* and *Flavonifractor*.

## Discussion

In the current study, we analyzed the cecal microbiota profile at different time points post–*S.* Enteritidis inoculation using 16S rRNA sequencing to elucidate temporal microbiota composition and the interaction between *S.* Enteritidis and cecal microbiota. The composition of the gut microbiome reflects co-evolution across the inhabiting microbes’ genetic, immune and metabolic interactions with the host [28]. High-throughput sequencing makes a composition-based microbial time series feasible by permitting analysis of temporal variations.

Development, genetics and *S.* Enteritidis inoculation contributed to cecal microbiome diversity. Age and developmental stage can have a significant impact on the microbiota richness and diversity [14]. In the current study, for the control group, the microbiota richness increased from 1 to 7 dpi and became stable after 14 dpi. Moreover, richness (Chao1) differed significantly between chickens at 3 and 7 dpi. The Simpson and Shannon indices were significantly different between 1 and 3 dpi, then become stable. However, it has been reported that Shannon diversity is significantly different between 2 and 7 dpi in the control group [12]. The different genetic background and development of chickens used could contribute to the different findings across time points in different studies. It has been reported that both richness and diversity are significantly higher in 6-week-old broiler chickens than in 1- or 3-week-old chicks [14], which is consistent with our results.

The microbial composition varies with development. *Firmicutes*, *Bacteroidetes* and *Proteobacteria* are the 3 most abundant phyla in ceca, respectively, representing 44–56, 23–46 and 1–16% of all taxa in the cecum [29], dominating microbiota composition in 1-week-old control chickens [12]. In the current study, in the control group, *Firmicutes* dominated microbiota composition from 1 to 35 dpi, followed by *Proteobacteria* (1 dpi) and *Bacteroidetes* (at 14–35 dpi); whereas in chickens inoculated with *S.* Enteritidis at 7 dpi, more-abundant *Firmicutes* was observed. *Firmicutes* followed by *Bacteroidetes* were the 2 most common phyla found in pigs after *S. typhimurium* infection [13]. Similar results have been reported previously [15]. The results of our PCA analysis indicated that *S.* Enteritidis inoculation moderately affected microbial community structure and composition in cecal content. Microbiome diversity was more affected by age than by treatment, which is consistent with previous results [7].

The chicken gut has two tasks that often interfere with one another: nutrient absorption and defense against pathogens. The microbial community plays an important role in maintaining normal physiological homeostasis, modulating the host immune system and influencing organ development and host metabolism [30]. Competitive exclusion (physical occupation, resource competition and direct physical or chemical insult to the potential colonist) is the main strategy by which gut microbiota exclude pathogens [31]. Normal microbiota contribute to the susceptibility of chicks to bacterial infection [32].

*S.* Enteritidis inoculation can affect the composition of the microbiome by changing the relative abundance of certain microbes. But the changes in cecal microbiota after *S.* Enteritidis inoculation were quite weak, which was similar to previous reports [15, 33]. Such inoculation significantly affected the abundance of microbiota at the genus level at each time point except for 1 and 21 dpi. It could take some time for *S.* Enteritidis to alter the abundance of cecal microbiota. Significantly different abundance in the microbiome at the genus level could be seen between the treated and control groups. The cecal microbiome community changes over time to protect the gut from *S.* Enteritidis inoculation. *Bifidobacterium*, *Rikenella*, *Coprococcus* and Lachnospiraceae *incertae sedis* played major roles in protecting against *S.* Enteritidis inoculation at an early stage (before 7 dpi), while *Bacillus*, *Blautia*, *Shuttleworthia* and *Flavonifractor* did so at a later stage (after 7 dpi; Additional file 4). Moreover, *Bacillus* positively correlated with *Blautia* and *Flavonifractor* (Fig. 12). It has been reported that older chickens are more resistant to *Salmonella* infection than are younger ones [34, 35], suggesting that gut microbiota play an important role in host resistance and the mature host immune system. Some studies also support this idea that early colonizers influence the relative abundance of the microbiome but the effect weakens over the long term [36].

We observed a greatly significant change in *Bifidobacterium* after *S.* Enteritidis inoculation. We assume that *S.* Enteritidis inoculation stimulates the immune system and *Bifidobacterium* proliferates as a biofilm to defend against pathogen infection. Increased *Bacillus* was also found in the current study (Additional file 4). It has been reported that *S.* Enteritidis can use organic acid produced by *Bacillus* as an energy source [37]. *Bacillus* appears only in the ceca of old chickens [10], which is identical to our finding that *Bacillus* significantly increased at 28 dpi.

*Blautia*, as a functional core group of intestinal flora, produces short-chain fatty acids by fermentation in the intestine; this benefits the host by lowering cecal pH value [38]. This can explain why *Blautia* content in the control group was higher than in the treated group only at late ages (28 and 35 dpi). The dramatic increase may benefit the host via resistance to pathogens. The volatile fatty acids produced by fermentation of the beneficial bacteria help control the amount of *Salmonella* in poultry [39].

## Conclusions

Our results indicated that both development and *S.* Enteritidis affect chicken cecal microbiota profiles. *S.* Enteritidis inoculation in young chicks also has effect on cecal microbiota, slightly reducing their diversity. *S.* Enteritidis inoculation influences the cecal microbiota mainly at 7 and 14 dpi; the relative abundance of these microbiota changed significantly after 14 dpi. The cecal microbiota exhibited immunity to *S.* Enteritidis inoculation at 28 dpi. Positive correlations between *Bacillus* and *Blautia* and between *Coprococcus* and *Flavonifractor* may benefit the chicken by providing resistance to *S.* Enteritidis.

## Additional files

**Additional file 1.** Data assessment and number of OTUs for each sample.

**Additional file 2.** Principal component analysis for samples in the control group.

**Additional file 3.** Principal component analysis for samples in the treated group.

**Additional file 4.** The differentially abundant genera between treated and control groups within each time point.
