# Supplementary figures and images for: Cecal microbiome profile altered by Salmonella enterica, serovar Enteritidis inoculation in chicken
Source: Gut Pathog. 2018 Aug 3;10:34. doi: 10.1186/s13099-018-0261-x (PMC6074038; doi:10.1186/s13099-018-0261-x)

# PCA

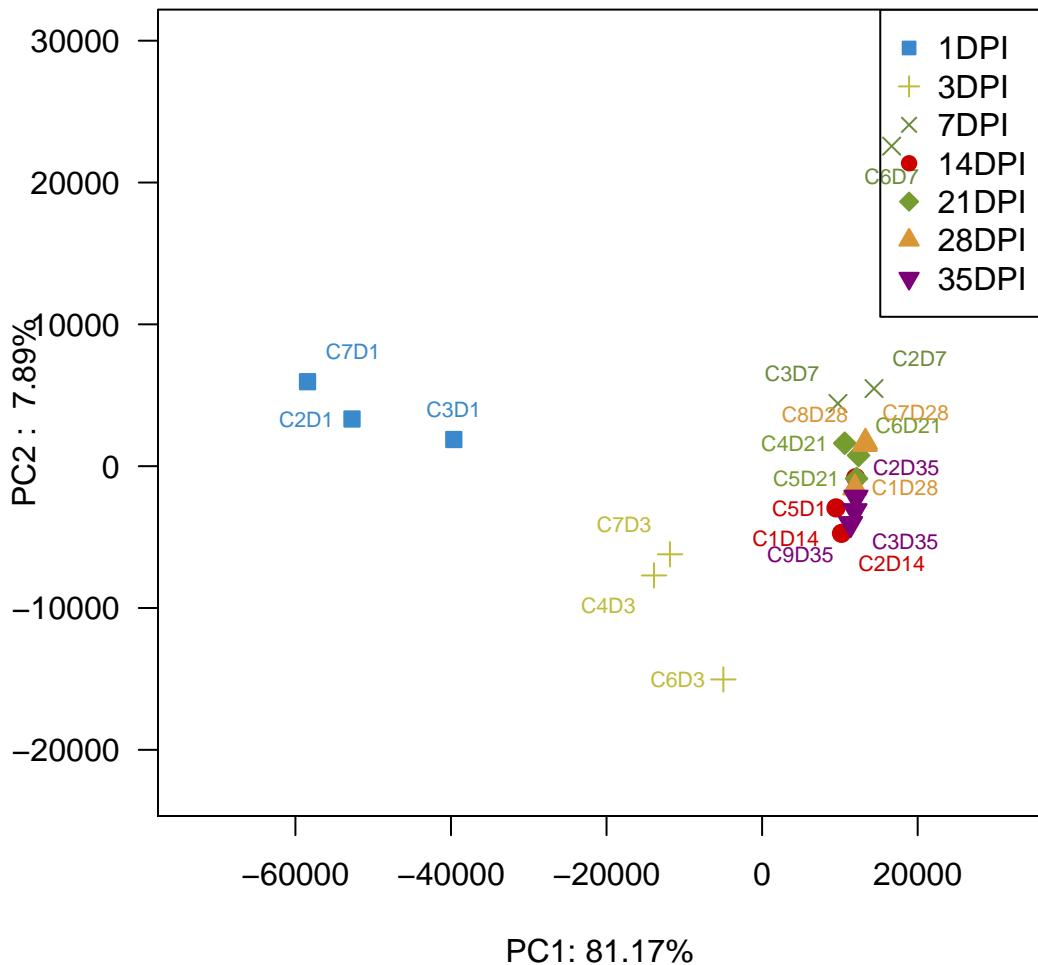

Supplement: Supplementary file 2 — Additional file 2. Principal component analysis for samples in the control group. [file 13099_2018_261_MOESM2_ESM.pdf]

# PCA

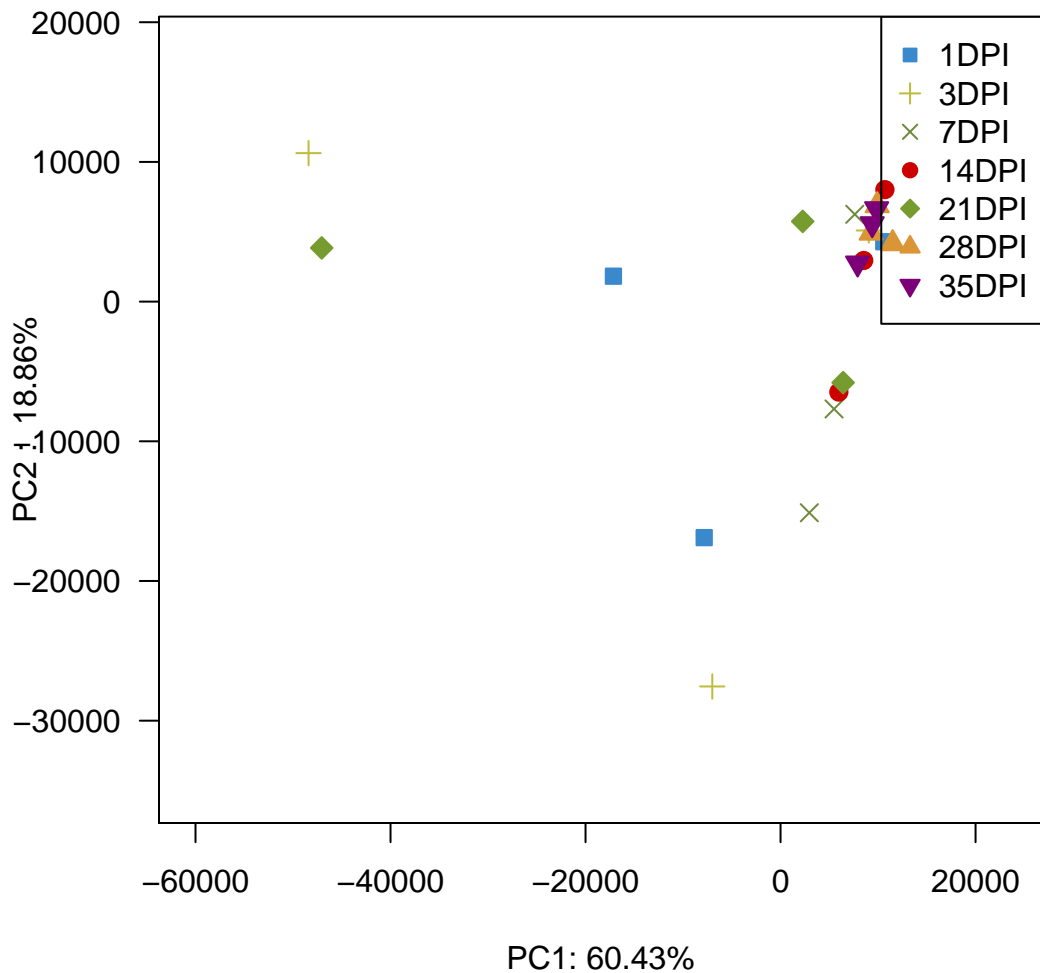

Supplement: Supplementary file 3 — Additional file 3. Principal component analysis for samples in the treated group. [file 13099_2018_261_MOESM3_ESM.pdf]
